# Supplementary material for: PEGR: a flexible management platform for reproducible epigenomic and genomic research
Source: Genome Biol. 2022 Apr 19;23:99. doi: 10.1186/s13059-022-02671-5 (PMC9016988; doi:10.1186/s13059-022-02671-5)
Supplement: Supplementary file 1 — Additional file 1. Integrated supplementary figures S1 and S2. [file 13059_2022_2671_MOESM1_ESM.pdf]

Supplementary Figures

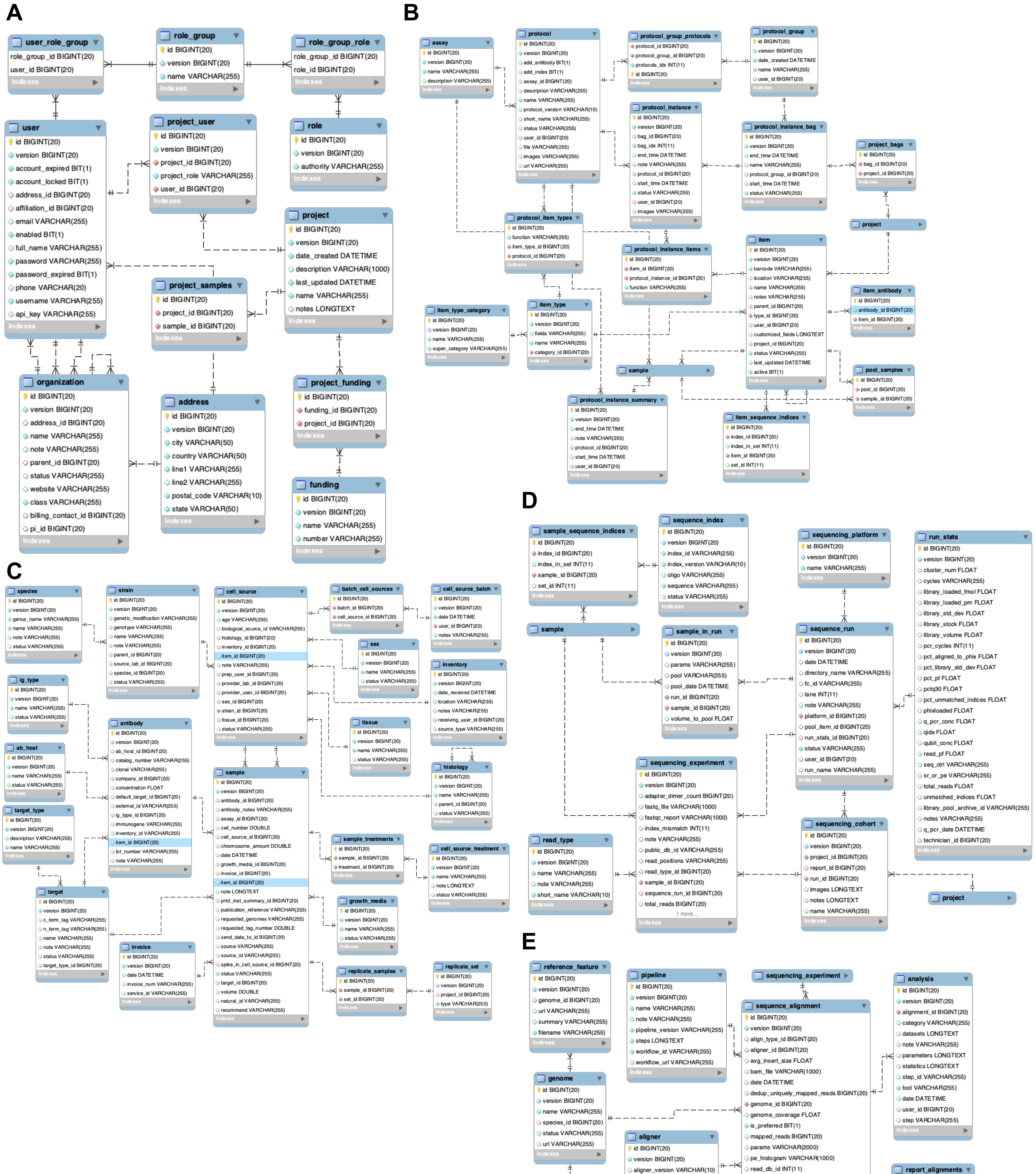

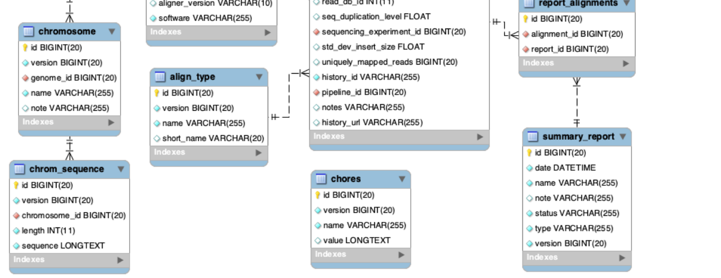

Figure S1: MariaDB table schematic underlying PEGR. (A) Relational structure for User roles in PEGR **(B)** Relational project structure **(C)** Relational structure for sample details **(D)** Relational structure for tracking sequencing runs relative to samples **(E)** Relational structure for tracking bioinformatic analyses and linking them to samples.

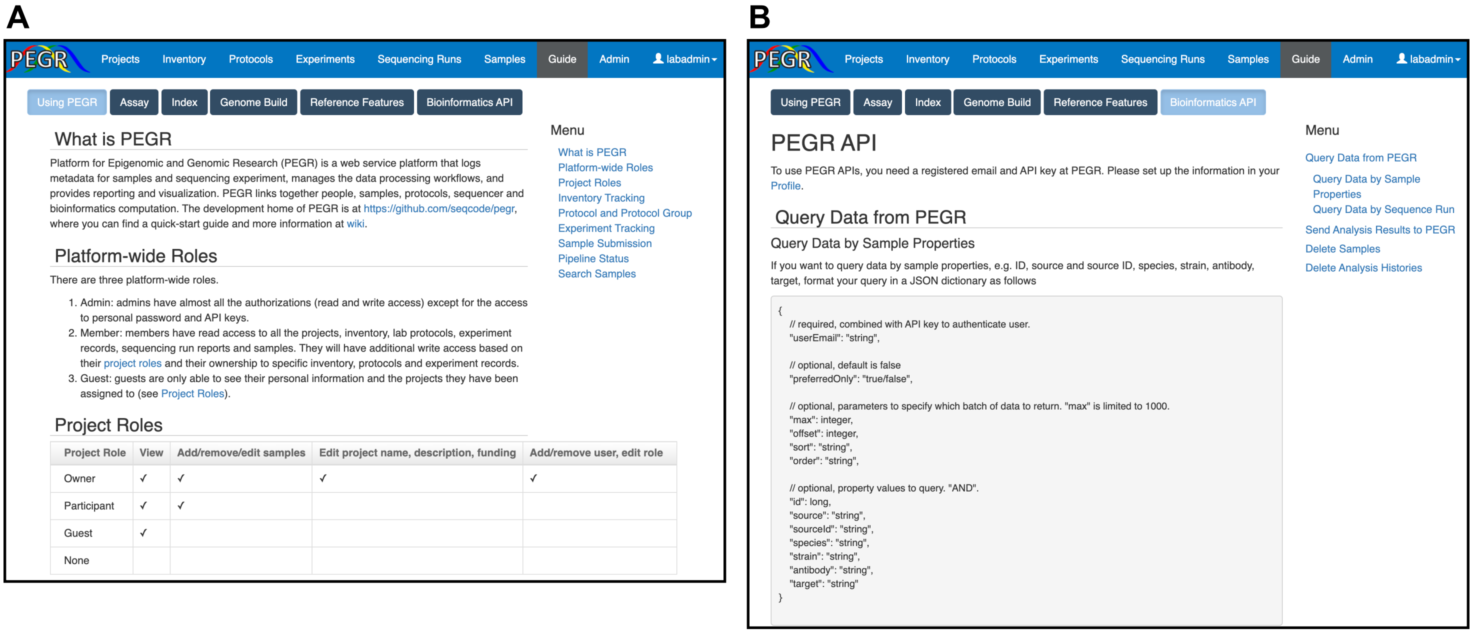

Figure S2: Guide to advanced PEGR operations. (A) The 'Guide' tab on the PEGR navbar provides a comprehensive breakdown on PEGR's structure and usage. **(B)** Details and sample code for accessing PEGR's RESTful API are provided here as well in two common programming languages (i.e., Java and Python)
